# Pittsburgh Landing

**Published:** 1862-05

**Authors:** 


### PITTSBURG LANDING.

Happily for us our experiences are the profits of life, which can be invested as other capital in improving ourselves or others.

Our Donelson experience gave ample opportunity for improvement in similar succeeding expeditions. Many unlooked for embarrassments owing to imperfect preparation were remedied, and our enterprise an entire success.

Our Sanitary Commission were telegraphed on Tuesday succeeding the battle for a supply of surgeons and nurses by Gen. Strong of Cairo. By 5 o'clock in the afternoon the following surgeons were on board the cars:

Drs. Ingals, Lynn, Isham, Ross, Amerman, Hamill, Davidson, Fisher, Bloodgood, Miller, Gillett, Allen, and myself.

Medical Students Mehler, Beasley, Zahn, Hollingsworth, and Annis.

Forty-eight nurses accompanied us, among others, Rev. Mr. Patton, Rev. Collyer, Messieurs Chadborn, Mason, Eddy, Judge Scates, etc.

We were sent in a special train, and made good time, nothing occurring to interfere with our progress or enjoyment of the trip. It was cheering as we left our cold and overflown prairie, to notice, as we progressed, the gradual change, marking our change of climate. The grass began to carpet the earth with its green tapestry, while the bursting beauty of the blossom covered peach tree, and the woods manting themselves in expanding foliage, were comforting changes to our northern eyes. While nothing did occur to interrupt our satisfaction, one of our surgeons came near being the subject of a hernial strangulation. He took off his truss upon retiring for the night, and inadvertently arose without reajusting it—and possessing a repletion of abdominal viscera he was soon reminded of his remissness. His truss was one of an unusual pattern, and required so much explanation that he was well nigh exhausted, and if his flagging powers had not been well sustained we might have had surgery nearer than Pittsburg.

We had also a fine specimen of magic—one of the finest and most ingenious performances I have ever seen. A gentleman took a shawl, rolled up in the usual travelling style, cylindrical bundle, and placing one end of it to his mouth imitated drinking from a bottle, and so perfect was the trick (the reader will be interested in knowing that the peculiarity of the performance was in the operator's deceiving himself instead of his audience,) that he imagined he had drank, and from everything we could see he felt so. The effect, also, so far as we could judge, lasted about the time of three fingers. This was repeated at intervals for the amusement of our company, with unfailing relish.

We arrived in Cairo at 9 o'clock, a. m., on Wednesday, and found it one degree lower in my estimation—that of complete submergence. A few buildings along the levee are all the exceptions to be made. Venice with the exception of the Gondola. A child was drowned the next Monday while going to call its father to dinner.

We breakfasted by an arrangement of the Commission, and for a short time, owing to the conflicting and perhaps unauthorized rumors of departure, we seemed about to be garroted a while with red terror. But by the exertion of Mr. Pulta and Dr. Isham, we were transferred at once on board the Louisiana, a hospital boat just landed from St. Louis. This was a most fortunate arrangement for us, for on it we were fitted fitted out with comfortable berths and good food; all arranged and paid for by our Commision.

The "Louisiana" was an elegant boat for the purpose, admirably fitted up, corps of surgeons and nurses, apothecary with complete assortment of medicines, wooden bedsteads with straw beds or matrasses on them, all clean, comfortable, and in good order.

The boat surgeon was Dr. Wagner, a very quiet, civil gentleman, a West-Pointer, but, in my estimation, lacking the least bit of an article we sometimes call "snap" at others "gituip."

His assistant was as opposite as possible, all energy, vivacity, bright, exceedingly interesting, and very well suited to the other man's place.

We had also our indefatigable Dr. Douglas, who gives every indication of being a man for the place he fills. He is gentlemanly, attentive, accommodating and industrious, qualifications pleasant to those having intercourse with him and satisfactory to his employers. We had also Dr. Simmons, the medical director for Cairo. He is another regular army officer, and discovers his love for the color red—though very kind and think is disposed to do what is right, yet it is plainly hinted by those around him that subservience is no mean merit. I must not say much, however, as I may need his favor, and must not hazzard it by speaking too plainly. I was pleased with all I saw of him.

Our boat had a large, beautiful, yellow flag floating at the bow, the same used by all hospitals. Some fears were entertained of being fired into by guerilla marauders, but if there were any such near our flag, it protected it as it has done other boats since, while those not thus protected have been assaulted.

As we passed up the river, we began to notice the absence of enterprise—in bad buildings, no comfortable dwellings or convenient outhouses—and scarcely an orchard from the Ohio river to Pittsburg landing did we see.

About 10 o'clock, we met the "City of Memphis," the same I came down on on my former trip, loaded with wounded. She was the first boat down with wounded. We asked her how many she had on board, and received the fearful answer *nine hundred and eight.* It would have made my heart sick had I believed it, but it seemed so improbable that by common consent we conceded it to be a mistake of *five hundred.* But we ascertained at Mound City, on our return, that she actually landed *seven hundred and twenty* in the Mound City Hospital. When others went home and were sent other places. We thought when we had two hundred and thirty that we were much crowded on the same boat—but multiply that by four, and when and how they lived is more than I can interpret. They had tents erected on the hurricane deck, tarpaulins spread on the sides, and every possible foot of room every where occupied.

This seeming barbarity was rendered more satisfactory when we recollect that on the battlefield there was absolutely no acmodation for many—and that while to suffer intolerable crowding together, it was only for twelve hours, and they would be placed in comfortable and beautiful accommodations.

We met two others during the night, but neither was so heavily loaded.

During our passage, the two members of the Sanitary Committee accompanying us, Mr. Ptton and Dr. Isham, allotted to each surgeon his quota of nurses, who are to accompany him and be subject to his direction. This very much facilitated all our movements, and enabled us to work systematically and efficiently, and see that the curiosity hunters were soon satisfied.

We landed at our destination, Pittsburg Landing, Friday, at 4 P. M., forty-seven hours after leaving Chicago. This was more than gratifying to us when we found the great need of our services. I spoke of the benefits of experience in the begining of this, and this impressed itself on me at Donelson—that whem temporary assistants are needed at all, they are needed *at once*, and our Sanitary Committee should have everything organized in advancc for such occasions, especially their transportation, so that they may be on the ground when most needed, before others arrive, and while the injuries received need most attention.

Our delegation had the honor of being the first voluntary organization represented there—though seven hundred miles from it.

Upon arriving our Commission went on board the "Tigress," Gen. Grant's head quarters, to confer with the Medical Director, Dr. Murray, and after some consultation we were assigned to differenτ places on boats, and some to labor on the field among the tent hospitals.

Drs. Lynn, Ingals, Millar, Gillet and myself, were asigned to the "Hiawatha," with medical students Beasley and Mehler.

It had been raining all morning and was then pouring down rapidly, and when we undertook to pass on shore from our boat to the "Hiawatha it seemed a realization of some of the hardships to which our poor soldiers are exposed—infinite mud.

The constant passing of wagons, horses, artillery and men, with the incessant rain makes travel almost impossible, and there were horsemen and footmen, and wagons loaded and unloaded picking their way through endless mud of untold depths. Some balky mules were giving variety by allowing their drivers a chance at their epithetical vocabulary. Some had run out and began at the beginning again, and the mules still looking at them.

Footpaths are made along the hill-side above the wagon track, and it was a touching sight to see there, rolled in their blankets, bodies of several soldiers who had died on the boats and been brought and laid there, with their open glazed eyes looking up towards heaven, from which torrents of descending rain saturated their blankets and filled their sunken sockets, each passer by stepping over them, no kind friend to protect or care for them, even the natural ties of friendship being shivered by the stern necessities of war.

We found the landing paved for the last few yards of our way with beautiful sacked corn, which was not being improved by its position.

Upon arriving at our boat, we found it occupied by about sixty officers, who had been wounded and brought on it from the field, converting it, without any calculation, from a transport into a hospital boat.

There were twenty-five privates. The boat was under charge of Dr. Roskiton, of Gen. McArthur's Division. He is a German, and it is with some reluctance that I express my surprise that such a man can obtain the position he occupies. It convinces me that favoritism forwards more than merit. So far as I could judge, and it was the universal opinion of my companions as well that he did not possess a single qualification for the place he filled. He was unable to get his outfit of stores, had the patients lying in utter confusion, and the only operation I saw him perform, was trying to squeeze pus from an edematous eyelid. When it was found by the medical director that he was incapable of taking care of himself even—he added insult to injury by sending a Dutch assistant surgeon to superivse him.

We received occasional accessions of wounded that night until we numbered about one hundred. After arranging our patients, and dressing their wounds, Mr. Collyer and myself went aboard the "Chancellor" lying along side of us. The scenes there could not be described, in their true character were all the adjectives in our language applied to them. Men with every conceivable injury, and suffering with both pain and hunger, with nurses worn out, no attending surgeon, the boat being a Commisary boat, converted, as was our own, by force of circumstances to surgical uses, a hospital steward doing for them the best he could—numbering over three hundred, laid in every possible, except comfortable, position, on boxes, barrels, staves, sacks, on the hard floors, with wounded, mangled limbs resting on the uneven surface of a row of barrels, some resting on the guards of the boat, with no tarpaulin to keep out the drifting and drenching rain; others on the lower deck, almost covered in mud, among the boilers, in the dark, no one to attend, with any certainty, to their wants or sooth their sufferings, very many in the most exquisite agony, resting on the hard floor, with nothing but a single blanket to shield their fevered and aching bodies, from the unfeeling boards. And yet, in the midst of all this, you seldom hear them complain. A soldier must be very severely wounded to make any noise. In most cases a real cheerfulness is present. We took some wine and gave it to them, and labored for some time to get them as comfortable as possible.

The next day (Saturday), found us with our complement of wounded, near three hundred. We managed to get part of our army stores on board, and so far as any one of us could see, were ready to go. But it seemed almost impossible to to get away. I asked our boat surgeon what was keeping us, why not start Saturday night? He said the pilot did not want to start at night, and besides, *he had not enough wounded, if he started with so few, charges would be preferred against him.* At that time every available point was occupied, except on the hurricane and boiler decks.

In that condition we stayed from Saturday until Sunday noon, with our two Dutchmen spinning arounnd each other, as if practicing a new edition of the lancers, until Director Simmons came aboard, and I asked, what in humanity's name we were waiting for?—why these men, wounded and suffering might not as well be lying in the Hospital at Mound City as in that boat? He could give no answer for the delay, except the stupidity of our asinine Dutchmen. I wish I could letter their hats. I would substitute U. A. S. for the ones I saw them sporting. He gave me the order, I carried it to the captain, and we were off. On Sunday we had a very agreeable and important addition to our stores from the Cinic. Sanitary Committee's boat. Very many things we were absolutely and hopelessly destitute of were kindly supplied by them, and we will not soon forget the kind generosity of their purveyor, Mr. Griffin, I think his name was. I was intrusted with the surgical management of the boat, and divided it into wards, assigning each surgeon his place with his nurses.

In my last letter I felt in duty bound to express an opinion relative to female army nurses. I shall never change it, but the place then suggested as proper to be filled by females, had my typical idea of all that is true and noble to fill it for our boat. I refer to Miss Safford, of Cairo. She embodies my idea of what a female should do and be, to accomplish her mission as an assistant to care for wounded men. She is active, kind, sympathizing, disinterested, prudent and indefatigable. She came aboard our boat, and we entrusted her with our cookery. It was a most trying place to come, where there was nothing, and organize and set in motion machinery that should supply the hungry wants of three hundred, and diver sify it to suit the capricious appetites of sick men. So well did she succeed, that our men had the next meal after she came on board at meal time. And so from day to day, everything worked by her management smoothly and perfectly, and it seemed the crowning glory of our mission to see this difficult part of hospital service so admirably rendered. I regard her as the Florence Nightingale of this war.

The kind of food served out by the Commissory, is, of course, ill fitted for use of sick men, and the supplies of delicacies from our Sanitary Committee and Cinics came in most excellently. The hard bread is a very serious draw-back to good living, and many a jest goes off at the expense of the well named bread. It is said to have been in service as roofing and pavement for six months without moistening through. One soldier of an antiquarian turn of mind, declared that one box marked B. C., meant *Before Christ.* Another converted it to a hitherto untried use. His leg was broken, and there being no splints, the surgeon found he had bandaged his up, using a *hard cracker as a splint!*

This last is really true, and a soldier witnessing the operation, cried out "Bully for the bread." We had an addition of three surgeons to our corps before leaving Drs. Roberts, Owen and Hamilton, all, I think, from Carbondale.

The class of cases we had on board were not so serious as those on my former trip. Our wounded were brought mostly from other boats, and the lighter cases were selected for moving, though many of them were severe enough. One young man was brought on in *articulo mortis*, and one Capt. Stevens, of Dixon, died. His leg had been amputated, and I am sorry to say did not appear to have been performed according to the light that surgeons of this age should have before them. He was a noble fellow, was suffering at the time of the battle with dysentery, but would not stay behind, no doubt, the depressed state of his constitution helped to secure the final result. We had on our boat the gallant Col. Moore, of the 21st Mo., who had also lost his right leg. There was a slight tendency to secondary hæmorrage in his case, but it had amounted to nothing when we left him at Mound City.

The amputations showed a remarkable atonic tendency. None seemed to take on a vigorous healing character. One fine intellectual-looking German, with an amputated limb, was constantly delirious. There seemed no reason, so far as appearances went, why he should not get well, yet he went slowly down and was in *articulo mortis* when we left him. One case of a German interested me. He was shot under the lower extremity of the left scapula—and I took out the battered ball between the lower border of the larnyx and the right sterno mastoid muscle, directly in front of the vessels. He had spit blood at first and suffered pain, but now seemed to be doing well, eating every meal. When he was asked if ever he had seen fight before, he turned up his shirt, and his scarred body needed no confirmation.

We found many wounded in the lower extremities, some accounted for it by the repeated order of Gen. Beauregard "to fire low—never kill when they could cripple—that it required two well men to care for each wounded one, while dead men took care of themselves."

The battle of Pittsburg Landing was eminently disastrous. I could calculate from the number on various boats and in Savanah, nearly six thousand, and how many more are in tents and more slightly wounded I had no means of ascertaining. There were in Savannah, eight miles below, for the first few days after the fight, twenty-one hundred scattered in twenty different places; all the houses, churches, or places capable of holding them being occupied. These were supplied with seven surgeons. Imagine the services rendered to three hundred freshly wounded men by one worn out surgeon?

The surgeons on our boat worked nobly, and their labors accomplished every thing anticipated. Our medical students had a rich treat, and a surgical feast.

There was but one surgeon who failed in his trust, and he certainly deserved all the contempt he received. He constantly presented himself at the apothecary desiring stimulants for his patients, which they as regularly did not get. This continued until he was drunk, suspicion was aroused, he detected drinking it, admonished, and subsided. I have no desire to injure the feelings of this man or his friends, but would say that if he cannot control himself he had better not sell the birth-right of his manhood quite so cheap. I withhold his name, suffice it, he was *not* from Chicago.

The arrangements for the care of the wounded are perhaps as good as can be under the circumstances. The Medical Directors are all complained of, and it is interesting to notice what efforts they make to convince you how entirely the whole army is dependent on them. "Why," said one of them to me, "I cannot get anything to do with, why, sir, all I have is obtained at the point of my sword." They are the most persecuted and self-sacrificing men in all the world if they don't deceive themselves.

The system of temporary volunteer aid adopted by Sanitary Commissions and States, undoubtedly has its great merits, and it is difficult to see how it could be superceded. Almost anything good can be diverted to unprofitable ends. It has been very plainly insinuated that on such occasions, when surgeons are temporarily given the oportunity of using the knife, that the opportunity is seized and appropriated to their own instead of their patient's benefit. It would not do to go home without doing an operation, and conservative surgery is lost sight of.

There is, of course, an abundance of surgery to be performed, and where they are competent there is no reason why volunteer surgeons should not relieve those who are worn out. On one boat the amount of surgery to be done was only limited by the endurance of the surgeons, and yet I am acquainted with and have every confidence in the gentlemen on board. Yet I have seen some very bad surgery done. There should be some competent man on every boat, and in every hospital, who should be held responsible to the Director for the conduct of his assistants. Many who go are reckless, and would rather have the credit of having amputated a leg than save the life of the best man in Christendom.

Our boat left Pittsburg Landing at noon on Sunday, and arrived at Mound City Hospital on Monday, at 2 o'clock, P.M. We had a few slight cases of Erysipelas on board, but none of any severity. The weather being mild, we kept our cabins ventilated, no odors accumulated, and we escaped. Our men were cheerful and grateful, and when we parted from them gave us pleasing evidence of their appreciation of our priceless labors.

CHICAGO, April 20th, '62. R**.

